# Predicting Ki-67 expression levels in non-small cell lung cancer using an explainable CT-based deep learning radiomics model

**DOI:** 10.3389/fonc.2025.1655714

**Published:** 2025-12-10

**Authors:** Shize Qin, Qing Jia, Chunmei Zhang, Man Li, Xiufu Zhang, Xue Zhou, Dan Su, Yongying Liu, Jun Zhou

**Affiliations:** 1 Department of Radiology, Jiangjin Central Hospital of Chongqing, Chongqing, China; 2 Chongqing General Hospital, Chongqing University, Chongqing, China; 3 Department of Pathology, Jiangjin Central Hospital of Chongqing, Chongqing, China; 4 Department of Research and Development, Shanghai United Imaging Intelligence Co., Ltd., Shanghai, China

**Keywords:** radiomics, deep learning, interpretability, non-small cell lung cancer, Ki-67 expression levels

## Abstract

**Objective:**

To predict Ki-67 expression levels in non-small cell lung cancer (NSCLC) using an interpretable model combining clinical-radiological, radiomic, and deep learning features.

**Methods:**

This retrospective study included 259 NSCLC patients from Center 1 (training/validation sets) and 112 from Center 2 (independent test set). Patients were grouped by a 40% Ki-67 cutoff. Radiomic features and deep learning features were extracted from CT images, where the deep learning features were obtained via a deep residual network (ResNet18). The least absolute shrinkage and selection operator (LASSO) was used to select optimal features and compute radiomics (rad-score) and deep learning (deep-score) scores. Univariate and multivariate logistic regression were used to identify independent clinical-radiological predictors of Ki-67. Four support vector machine models were developed: a clinical-radiological model (based on independent clinical-radiological features), a radiomic model (using the rad-score), a deep learning model (using the deep-score), and a combined model (integrating all the above features). SHapley Additive exPlanations (SHAP) analysis was used to visualize feature contributions. Models’ performance was assessed using receiver operating characteristic (ROC) curves and the integrated discrimination improvement (IDI) index.

**Results:**

High Ki-67 expression occurred in 76 (42.0%), 38 (48.7%), and 33 (29.5%) patients in the training, validation, and independent test sets, respectively. In the independent test set, the combined model achieved the highest predictive performance, with an AUC of 0.892 (95% CI: 0.828–0.956). This improvement over the clinical-radiological (0.820, 95% CI: 0.721–0.918), radiomics (0.750, 95% CI: 0.655–0.844), and deep learning (0.817, 95% CI: 0.732–0.902) models was statistically significant (all p<0.05), as supported by IDI values of 0.115, 0.288, and 0.095, respectively. SHAP analysis identified the deep-score, histological type, and rad-score as key predictors.

**Conclusion:**

The interpretable combined model can predict Ki-67 expression in NSCLC patients. This approach may provide imaging evidence to assist clinicians in optimizing personalized therapeutic strategies.

## 1 Introductions

Non-small cell lung cancer (NSCLC) is the most common pathological type of lung cancer and is associated with a relatively low average five-year survival rate (1–4). This clinical reality highlights the urgent need for improved prognostic assessment. Therefore, Ki-67, a validated indicator of tumor cell proliferation, has gained significant attention (5). Its expression level is not only closely linked to the aggressive behavior of NSCLC (6, 7) but also serves as a critical prognostic determinant, with high Ki-67 expression being associated with markedly shorter progression-free and overall survival periods (8, 9). Furthermore, research has demonstrated the Ki-67 index may be clinically significant for predicting neoadjuvant chemotherapy effectiveness in stage I-IIIA NSCLC and chemotherapy responses in advanced NSCLC (10, 11). Currently, Ki-67 expression is typically assessed using immunohistochemistry (IHC); however, this method faces two key challenges: its invasive nature (12), and the inability of a localized sample to represent the entire tumor, given NSCLC’s significant heterogeneity (13, 14). Thus, a non-invasive method for characterizing tumor heterogeneity is needed for the accurate evaluation of Ki-67.

Radiomics addresses this by quantifying image-based features, which correlate with histopathology and show potential for Ki-67 prediction (15, 16). However, such predefined features are intrinsically limited in capturing the complex NSCLC microenvironment (17, 18). In contrast, deep learning can automatically extract high-level features directly from images, capturing complex information inaccessible to handcrafted radiomics (19, 20). The study further indicates that integrating radiomic and deep learning features can improve the classification of lung cancer subtypes and prognosis while achieving multi-modal information fusion (21, 22).

Therefore, this study aims to develop a model combining clinical-radiological, radiomic, and deep learning features to predict Ki-67 expression levels. We further use the Shapley Additive Explanations (SHAP) technique to interpret model outputs, with the goal of providing imaging evidence for personalized treatment planning.

## 2 Methods

### 2.1 Patient population

This retrospective study was conducted in accordance with ethical standards and was approved by the institutional review boards of [Jiangjin Central Hospital of Chongqing (Center 1)] (Approval No: KY20241204-001) and [Chongqing General Hospital (Center 2)] (Approval No: KY S2024-058-01), with a waiver of informed consent. It analyzed patient data from Center 1 and Center 2 between June 2022 and September 2024.

Inclusion criteria (1): Pathologically confirmed NSCLC by surgical resection or biopsy, with documented Ki-67 IHC results (2); Chest CT scans obtained before biopsy or surgery. Exclusion criteria (1): Patients with poor CT image quality or incomplete clinical/imaging data (2); Patients with unclear delineation between lesions and adjacent obstructive pneumonia or atelectatic tissue (3); Patients who received any treatment or any invasive examination before CT examination.

**Figure 1:**
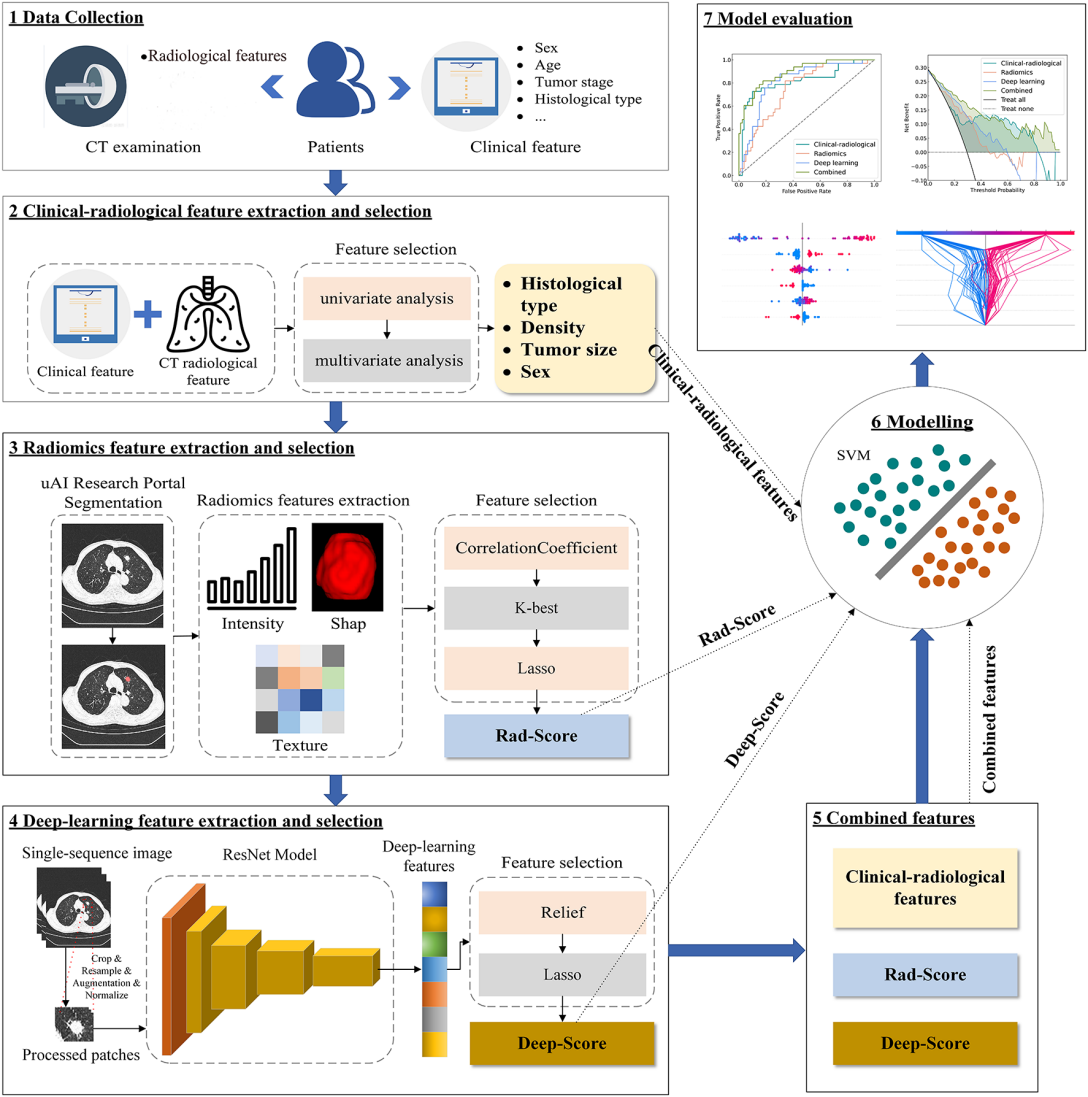
Technical roadmap.

A total of 259 patients were enrolled from Center 1 (163 males, 96 females, mean age 65.4 ± 9.7 years), randomly divided into a training set (N = 181) and a validation set (N = 78) at a ratio of 7:3. At Center 2, an independent test set was established with 112 patients (60 males, 52 females, mean age 65.5 ± 9.6 years). The overall study design and analytical pipeline are summarized in **Figure 1**.

### 2.2 Pathological assessment

IHC analysis was used to assess Ki-67 expression, defined as the percentage of positively stained cells. Under high magnification (*400), the number of Ki-67 positive tumor cells was quantified; Ki-67 expression was calculated as: (number of positive tumor cells in each zone/total number of tumor cells in each zone) ×100%. Values from five different areas were recorded and averaged. Previous studies have reported significant prognostic differences in NSCLC patients when a 40% cutoff value for Ki-67 expression is used (23–26). Furthermore, several radiomics studies aiming to predict Ki-67 levels have also adopted this 40% threshold (27, 28). Thus, this study defined high Ki-67 expression as ≥40% and low expression as <40%.

### 2.3 CT examination

CT examination was performed using two scanners: a UCT530 40-row helical CT scanner (United Imaging Healthcare, Shanghai, China) at Center 1, and a Philips IQon Spectral 64-row helical CT scanner (Philips Medical Systems, Best, Netherlands) at Center 2. The scanning parameters were configured as follows: Center 1: Tube voltage of 120 kV, automatic tube current modulation (reference: 80 mAs), pitch factor of 1.075, rotation time of 0.6 s/rev, collimation width of 22 mm, acquisition matrix of 512×512, reconstruction slice thickness/interval of 1 mm, and lung window settings of window width 1200 HU/window center -600 HU; Center 2: Tube voltage of 120 kV, automatic tube current modulation (reference: 129 mAs), pitch factor of 1.000, rotation time of 0.5 s/rev, maintaining identical collimation width (22 mm), acquisition matrix (512×512), reconstruction parameters (1 mm thickness/interval), and lung window settings (window width 1200 HU/window center -600 HU).

### 2.4 Clinical data collection and CT radiological feature evaluation

Baseline clinical data of patients were systematically documented, encompassing demographic characteristics (gender, age, alcohol consumption, smoking history), tumor profiles (clinical stages, histological type), clinical symptoms (cough, sputum production, fever, breathing difficulty, chest tightness, headache, lymphadenopathy), and laboratory data (white blood cell count, lymphocyte count, platelet count, hemoglobin level, red blood cell count, creatinine level).

Two radiologists independently assessed the CT radiological features in a double-blind method, which were defined as expert-based descriptions of lesion morphology. The assessed features included tumor location, shape, maximum cross-sectional diameter, bronchial cutoff sign, lobulation, air bronchogram, spiculation, pleural tag, vacuole (or cystic component), and tumor density. Any disagreements were resolved by consensus.

### 2.5 Image segmentation

The uAI Research Portal (29) (version: 20240730, https://urp.united-imaging.com/; Shanghai United Imaging Intelligence Co., Ltd., Shanghai, China), a licensed commercial platform, was employed for image segmentation and all subsequent classification modeling. This one-stop analysis system for multimodal clinical research images was developed using Python (version 3.7.3) and incorporated the widely used PyRadiomics package. Access to the platform was granted under our institutional license agreement with the vendor. Using the platform’s integrated VB-Net model, regions of interest (ROIs) were automatically segmented and reconstructed into 3D volumes of interest (VOIs). This model, previously validated for pulmonary nodules [average Dice similarity coefficient (DSC): 0.915] (30). Additionally, a three-tier arbitration mechanism was established: two radiologists with 5 and 8 years of experience, respectively, independently performed slice-by-slice corrections of the ROIs. The inter-observer DSC was 0.887. For regions with high inter-observer agreement (DSC ≥ 0.700), the contours from the more experienced radiologist (8 years) were used. In cases of inter-observer discrepancies (DSC < 0.700), a senior radiologist with over 15 years of experience arbitrated and finalized the contours.

### 2.6 Feature extraction and selection

#### 2.6.1 Radiomic feature extraction and selection

Before feature extraction, all CT images underwent a standardized preprocessing pipeline. This included isotropic resampling to a median voxel size of 0.7×0.7×1 mm³, gray-level discretization with a fixed bin width of 25, and setting the window width and level to 1500 HU and -600 HU, respectively. Radiomic feature extraction was performed on the segmented VOIs in compliance with the Image Biomarker Standardization Initiative (IBSI) guidelines. A total of 2264 features were extracted, which can be categorized into three groups: 104 shape-based features, 432 first-order statistical features, and 1728 second-order texture features.

Feature selection was exclusively conducted on the training set to avoid data leakage and overfitting, following a multi-step process (1): Z-score normalization of all features (2); removal of features with near-zero variance or high inter-correlation (Pearson’ r > 0.8) (3); selection of outcome-associated features via the SelectKBest method (F-statistic) (4); identification of the most robust, non-redundant feature set for modeling using the least absolute shrinkage and selection operator (LASSO) with 10-fold cross-validation.

The radiomics score (rad-score) was computed as a linear combination of the LASSO-selected features, weighted by their coefficients. The formula for the rad-score is as follows (Equation 1):

(1) $$Rad-score=\sum_{i}^{n}(Cofficient_{i}\times Feature_{i})+b$$

where *n* is the number of selected features, $Feature_{i}$ and $Cofficient_{i}$ are the Z-score standardized value and its corresponding LASSO regression coefficient for the i-th feature, respectively, and *b* is the intercept of the model

#### 2.6.2 Deep learning feature extraction and selection

A deep residual network (ResNet18) was implemented for feature extraction and trained from scratch, without using pre-trained weights or transfer learning. The original CT images were subjected to multi-stage preprocessing (see **Supplementary Text 1** for specific preprocessing procedures). The preprocessed images were fed into ResNet18, optimized with a hybrid loss function combining Focal Loss and Dice Loss. Focal Loss served as the primary classification objective, while Dice Loss—computed between class activation maps (CAM) and ground-truth lesion regions—guided the network to focus on lesion areas, thereby improving both interpretability and classification accuracy. The total loss is defined as (Equation 2):

(2) $$L_{loss}=L_{focal}+\alpha L_{dice\;}$$

where α is an empirically determined weighting coefficient (default α=0.05) that controls the relative contribution of the Dice loss.

The model was performed using the AdamW optimizer (β_1_=0.9, β_2_=0.999, weight decay=0.01) combined with a step-based learning rate scheduler (step size: 1000 epochs, decay factor: 0.1) for dynamic learning rate adjustment. Early stopping was employed to prevent overfitting. After training, the model with the highest validation AUC was selected as the final model for both end-to-end classification performance evaluation and deep feature extraction.

For deep feature extraction, the global average pooling layer was selected as the feature representation layer. Input CT images were preprocessed and fed into the finalized model (with frozen parameters), and the activations from the global average pooling layer were extracted as 256-dimensional feature vectors. Throughout this process, all model weights remained fixed without fine-tuning.

Deep learning feature selection comprised the following steps (1): Z-score normalization of all features (2); Application of the Relief algorithm to evaluate feature relevance (3); Final selection and weighting of the most discriminative features using LASSO regression.

The deep learning score (deep-score) was then computed as a linear combination of these selected features, weighted by their LASSO coefficients, following the formula (Equation 3):

(3) $$\text{Deep}-score=\sum_{i}^{n}(Cofficient_{i}\times Feature_{i})+b$$

where *n* is the number of selected features, $Feature_{i}$ and $Cofficient_{i}$ are the Z-score standardized value and its corresponding LASSO regression coefficient for the i-th feature, respectively, and *b* is the intercept of the model

### 2.7 Model development and validation

All modeling was conducted within the uAI Research Portal using the open-source Scikit-learn library (Scikit-learn 0.23.2). To ensure a fair comparison of predictive performance across feature modalities, clinical-radiological (based on independent clinical-radiological features), radiomics (using rad-score), and deep learning (using deep-score) models were developed with a uniform support vector machine (SVM) classifier. SVM is well-suited for handling high-dimensional data and potential non-linear relationships. For the combined model integrating clinical-radiological features, rad-score, and deep-score, the SVM classifier was compared against four other algorithms: Decision Tree, Random Forest, XGBoost, and Logistic Regression. The SVM demonstrated better performance on the validation set (**Supplementary Table S1**) and was consequently selected. All models were optimized for hyperparameters via grid search, with the specific parameters detailed in **Supplementary Table S2**.

Receiver operating characteristic (ROC) curves were plotted to calculate the area under the curve (AUC), specificity, sensitivity, and accuracy. Calibration curves, along with the Brier score, assessed the agreement between model predictions and pathologic outcomes. Decision curve analysis (DCA) was applied to quantify the clinical net benefit across a range of threshold probabilities from 0% to 100%.

### 2.8 Model interpretation

SHAP is an open-source python library (RPID: SCR_021362) for machine learning model interpretation. It quantitatively assesses the contribution of each feature to model outputs through Shapley value calculation. In this study, feature summary plot, beeswarm plot, decision plot and force plot were generated to provide global and local interpretations.

### 2.9 Statistical analysis

The Kolmogorov–Smirnov test was applied to assess data distribution normality. Normally distributed data were presented as mean ± standard deviation ( $\bar{\text{x}}$ ± s), whereas non-normally distributed data were reported as median with interquartile range [M (Q25, Q75)].

A univariate analysis was performed in the train set to assess each clinical and radiological feature’s association with the outcome. Continuous variables were compared using the student’s t-test or Mann-Whitney U test, while categorical variables were compared using the Chi-square test or Fisher’s exact test. Features with a p-value < 0.05 in the univariate analysis were subsequently included in the multivariate analysis. Multivariate analysis was conducted to identify clinical and radiological features independently associated with Ki-67 expression level. Model comparison between the combined model and the individual models was performed using the integrated discrimination improvement (IDI) index to quantify the magnitude of the net improvement in predicted probabilities. Statistical analyses were executed using the uAI Research Portal and SPSS statistical software (version 26.0, https://www.ibm.com). A *p*-value<0.05 was considered statistically significant.

## 3 Results

### 3.1 Clinical-radiological feature and clinical-radiological model

The clinical-radiological features of the three datasets are summarized in **Table 1**. IHC stratification showed high Ki-67 expression (≥40%) was present in 42.0% (76/181), 48.7% (38/78), and 29.5% (33/112) of cases in the training, validation, and independent test sets, respectively. Multivariate analyses identified histologic type (p<0.001), diameter (p=0.026), density (p=0.009) and gender (p=0.041) as independent clinical-radiological features predicting Ki-67 expression level (**Table 2**). The clinical-radiological model achieved the AUC values of 0.763 (training set), 0.803 (validation set), and 0.820 (independent test set), as detailed in **Table 3**.

**Table 1:** Clinical-radiological features of the training, validation, and independent test sets.

Clinical-radiological features	Training	Validation	Independent test	*P*
ki-67 expression level, n (%)				0.019
High-expression group	76 (42.0)	38 (48.7)	33 (29.5)	
Low-expression group	105 (58.0)	40 (51.3)	79 (70.5)	
Gender, n (%)				0.095
Male	109 (60.0)	54 (69.2)	60 (53.6)	
Female	72 (40.0)	24 (30.8)	52 (46.4)	
Age (M (Q25, Q75)), years	68 (58–73)	68 (58–72)	67 (58-72)	0.968
Alcohol consumption, n (%)				0.554
Heavy	30 (16.6)	14 (18.0)	13 (11.6)	
Light	51 (28.2)	20 (25.6)	27 (24.1)	
Never	100 (55.2)	44 (56.4)	72 (64.3)	
Smoking history, n (%)				0.713
Long-term	61 (33.7)	32 (41.0)	39 (34.8)	
Ever	30 (16.6)	9 (11.5)	15 (13.4)	
Never	90 (49.7)	37 (47.5)	58 (51.8)	
Clinical stage, n (%)				0.017
I	58 (32.0)	22 (28.2)	53 (47.3)	
II	26 (14.4)	5 (6.4)	10 (8.9)	
III	49 (27.1)	21 (26.9)	21 (18.8)	
IV	48 (26.5)	30 (38.5)	28 (25.0)	
Histological type, n (%)				0.178
Squamous cell carcinoma	51 (28.2)	26 (33.3)	24 (21.4)	
Adenocarcinoma	130 (71.3)	52 (66.7)	88 (78.6)	
Cough, n (%)				<0.001
Yes	124 (68.5)	46 (59.0)	48 (42.9)	
No	57 (31.5)	32 (41.0)	64 (57.1)	
Abundant sputum, n (%)				0.687
Yes	65 (35.9)	28 (35.9)	35 (31.2)	
No	116 (64.1)	50 (64.1)	77 (68.8)	
Fever, n (%)				0.648
Yes	7 (3.9)	4 (5.1)	7 (6.3)	
No	174 (96.1)	74 (94.9)	105 (93.7)	
Breathing difficulty, n (%)				<0.001
Yes	45 (24.9)	17 (21.8)	5 (4.5)	
No	136 (75.1)	61 (78.2)	107 (95.5)	
Chest tightness, n (%)				0.390
Yes	24 (13.3)	9 (11.5)	9 (8.0)	
No	157 (86.7)	69 (88.5)	103 (92.0)	
Headache, n (%)				0.160
Yes	9 (5.0)	4 (5.1)	1 (0.9)	
No	172 (95.0)	74 (94.9)	111 (99.1)	
Lymphadenopathy, n (%)				0.162
Yes	52 (28.7)	24 (30.8)	44 (39.3)	
No	129 (71.3)	54 (69.2)	68 (60.7)	
White blood cell count (M (Q25, Q75)),10^^^9/L	6.51 (5.06-7.85)	6.72 (5.09-8.07)	6.16 (5.11-8.11)	0.483
Lymphocyte count (M (Q25, Q75)),10^^^9/L	1.30 (1.03-1.57)	1.38 (1.00-1.66)	1.54 (1.20-1.89)	<0.001
Platelet count (M (Q25, Q75)),10^^^9/L	243 (197-290)	241 (190-292)	216 (181-285)	0.115
Hemoglobin level (M (Q25, Q75)), g/L	129 (117-139)	130 (120-137)	130 (120-139)	0.712
Red cell count (M (Q25, Q75)),10^^^12/L	4.26 (4-4.50)	4.26 (4-4.60)	4.33 (4-4.60)	0.828
Creatinine level (M (Q25, Q75)), umol/L	68 (60-76)	68 (59-81)	64 (57-74)	0.182
Tumor location, n (%)				0.373
Central type	19 (10.5)	9 (11.5)	7 (6.3)	
peripheral	162 (89.5)	69 (88.5)	105 (93.7)	
Tumor shape, n (%)				
Round/oval	19 (10.5)	3 (3.8)	5 (4.5)	0.065
Irregular	162 (89.5)	75 (96.2)	107 (95.5)	
Tumor diameter (M (Q25, Q75)), mm	34 (20-50)	31 (21-52)	26 (18-42)	0.028
Bronchial cutoff sign, n (%)				0.456
Yes	54 (29.8)	27 (34.6)	41 (36.6)	
No	127 (70.2)	51 (65.3)	71 (93.4)	
Lobulation, n (%)				0.765
Yes	166 (91.7)	73 (93.6)	105 (93.7)	
No	15 (8.3)	5 (6.4)	7 (6.3)	
Air bronchogram, n (%)				0.014
Yes	92 (50.8)	48 (61.5)	45 (40.2)	
No	89 (49.2)	30 (38.5)	67 (59.8)	
Spiculation, n (%)				0.271
Yes	151 (83.4)	66 (84.6)	86 (76.8)	
No	30 (16.6)	12 (15.4)	26 (23.2)	
Pleural tag, n (%)				0.117
Yes	161 (89.0)	75 (96.2)	98 (87.5)	
No	20 (11.0)	3 (3.8)	14 (12.5)	
Vacuole (or cystic component), n (%)				0.801
Yes	27 (14.9)	14 (17.9)	19 (17.0)	
No	154 (85.1)	64 (82.1)	93 (83.0)	
Tumor density, n (%)				0.095
Solid	139 (76.8)	62 (79.5)	72 (64.3)	
Partially solid	32 (17.7)	13 (16.7)	33 (29.5)	
Ground glass	10 (5.5)	3 (3.8)	7 (6.2)	

**Table 2:** Univariate and multivariate analysis results of clinical-radiological features.

Clinical-radiological features	Univariate			Multivariate		
	Coefficient	OR (95% CI)	*P*	Coefficient	OR (95% CI)	*P*
Lobulation	-2.446	0.087 (0.001-0.674)	0.019			
Tumor location	-1.225	0.294 (0.106-0.813)	0.018			
White blood cell count	0.170	1.185 (1.034-1.359)	0.015			
Clinical stag	0.317	1.373 (1.064-1.771)	0.015			
Pleural tag	-2.035	0.131 (0.029-0.582)	0.008			
Cough	-0.985	0.373 (0.190-0.736)	0.004			
Bronchial cutoff sign	-1.009	0.364 (0.189-0.701)	0.002			
Spiculation	-1.779	0.169 (0.056-0.507)	0.002			
Alcohol consumption	-0.794	0.452 (0.299-0.684)	<0.001			
Tumor density	-2.878	0.056 (0.013-0.237)	<0.001	-2.612	0.073 (0.010-0.519)	0.009
Smoking history	-0.823	0.439 (0.309-0.624)	<0.001			
Gender	-1.827	0.161 (0.079-0.327)	<0.001	-1.479	0.228 (0.055-0.942)	0.041
Tumor diameter	0.061	1.063 (1.042-1.085)	<0.001	0.037	1.038 (1.004-1.072)	0.026
Histological type	2.958	19.250 (7.891-45.961)	<0.001	2.071	7.932 (2.633-23.895)	<0.001

**Table 3:** Performance of each model for predicting ki-67 expression level.

Model	Data set	AUC (95%CI)	Sensitivity	Specificity	Accuracy	F1-score
Clinical-radiological	Training	0.763 (0.689-0.837)	0.592	0.819	0.724	0.643
	Validation	0.803 (0.703-0.903)	0.711	0.775	0.744	0.730
	Independent test	0.820 (0.721-0.918)	0.727	0.886	0.839	0.727
Radiomics	Training	0.817 (0.756-0.878)	0.776	0.714	0.740	0.715
	Validation	0.784 (0.680-0.887)	0.711	0.725	0.718	0.711
	Independent test	0.750 (0.655-0.844)	0.515	0.772	0.696	0.500
Deep learning	Training	0.912 (0.869-0.955)	0.882	0.790	0.829	0.812
	Validation	0.800 (0.697-0.903)	0.763	0.725	0.744	0.744
	Independent test	0.817 (0.732-0.902)	0.818	0.696	0.732	0.643
Combined	Training	0.929 (0.892-0.966)	0.882	0.857	0.867	0.848
	Validation	0.825 (0.733-0.917)	0.737	0.800	0.769	0.757
	Independent test	0.892 (0.828-0.956)	0.788	0.823	0.812	0.715

### 3.2 Prediction performance of the radiomics model

**Figure 2:**
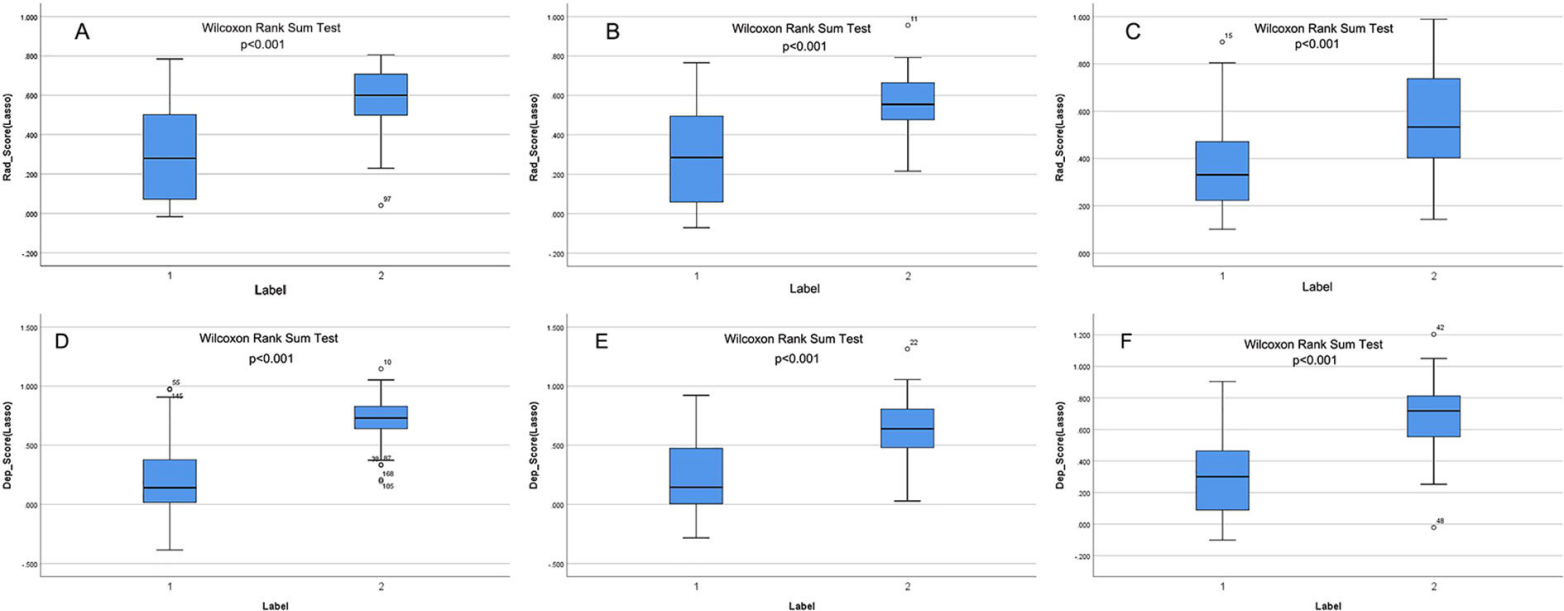
**(A–F)** show the rad-score and deep-score in training, validation, and independent test sets, respectively. Label 1 and Label 2 indicate the low- and high-expression groups.

Following feature selection, four robust radiomics features were retained to compute the rad-score (**Supplementary Table S3**). The high-expression group exhibited a significantly greater rad-score compared to the low-expression group across all three cohorts (training, validation, and independent test; all p<0.001), as shown in **Figures 2A–C**. The radiomics model achieved AUC values of 0.817 in the training set, 0.784 in the validation set, and 0.750 in the independent test set (**Table 3**).

### 3.3 Prediction performance of the deep learning model

**Figure 3:**
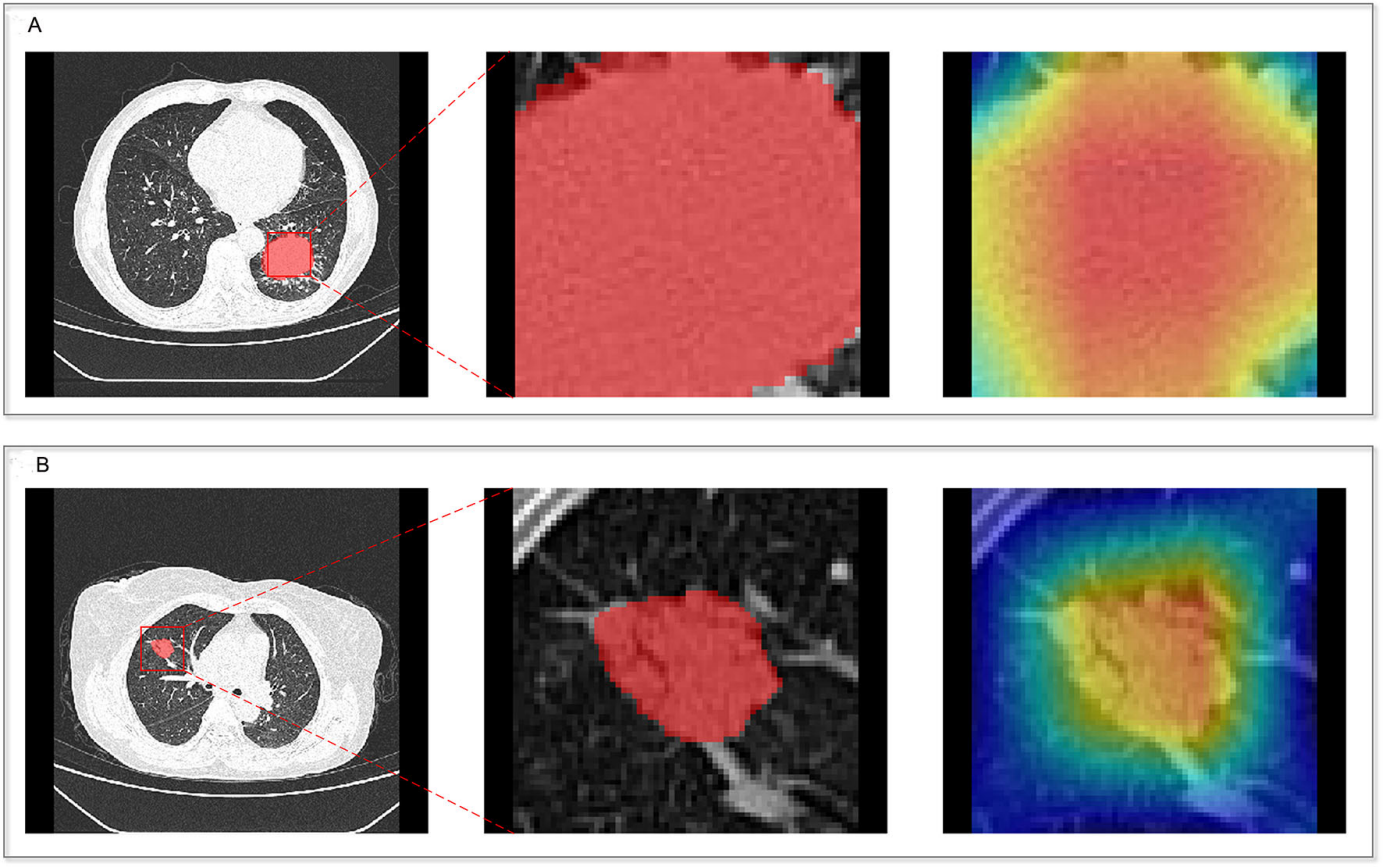
Heatmaps of the deep learning network for two patients. **(A)** Patient 1; **(B)** Patient 2.

The CAM module was used to visualize the attention regions that the deep learning network focused on when making classification decisions (**Figure 3**; a detailed explanation is provided in **Supplementary Text 2**). Four discriminative deep learning features were retained to compute the deep-score (**Supplementary Table S4**). The high-expression group exhibited a significantly greater deep-score compared to the low-expression group across all three cohorts (all p<0.001), as shown in **Figures 2D–F**. The AUCs of the training, validation, and independent test sets of the deep learning model were 0.912, 0.800, and 0.817, respectively (**Table 3**).

### 3.4 Prediction performance of the combined model

**Figure 4:**
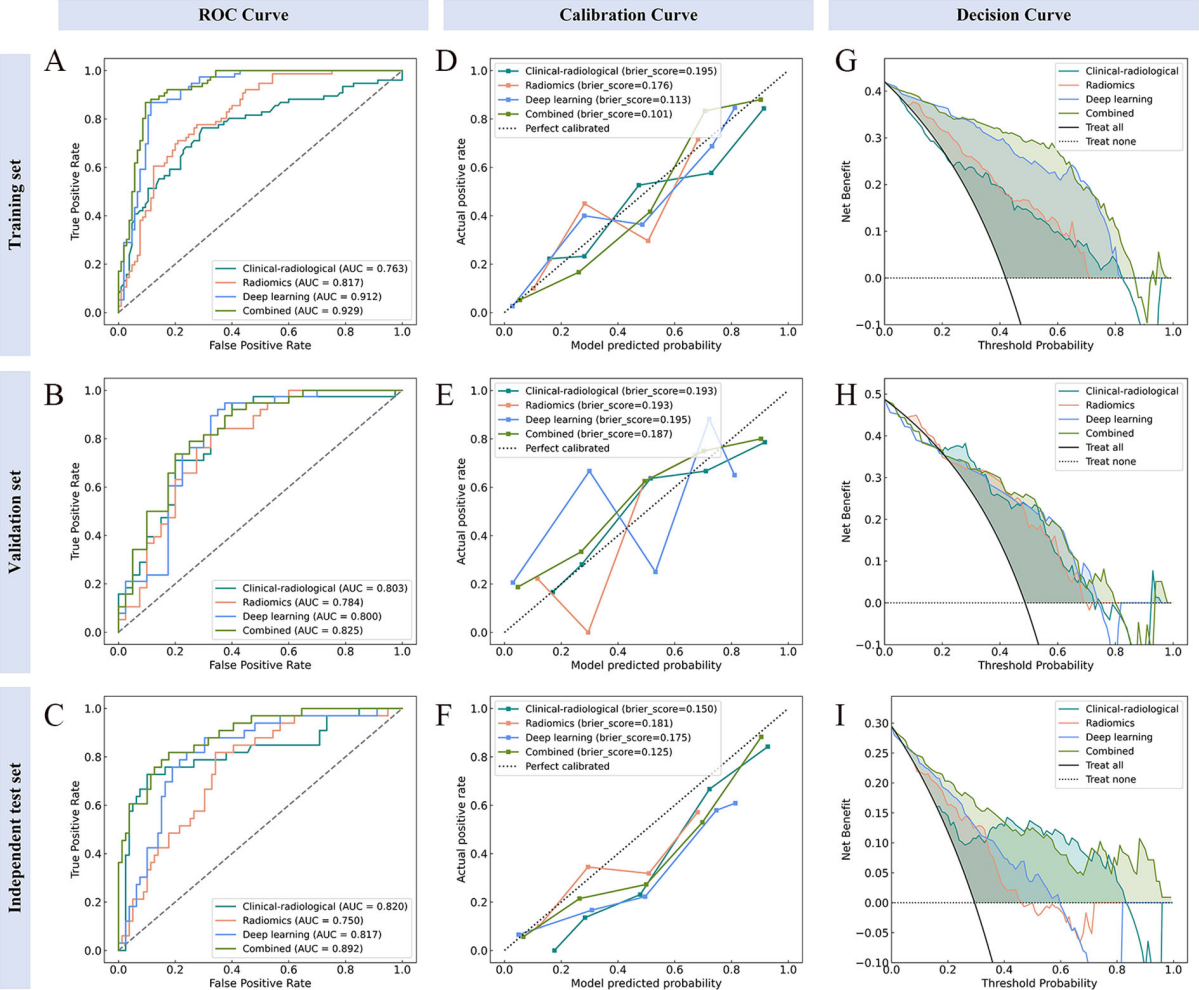
Performance evaluation of the model across different datasets. ROC **(A–C)**, calibration **(D–F)**, and decision curve analysis **(G–I)** curves for the training, validation, and independent test sets, respectively.

**Figure 5:**
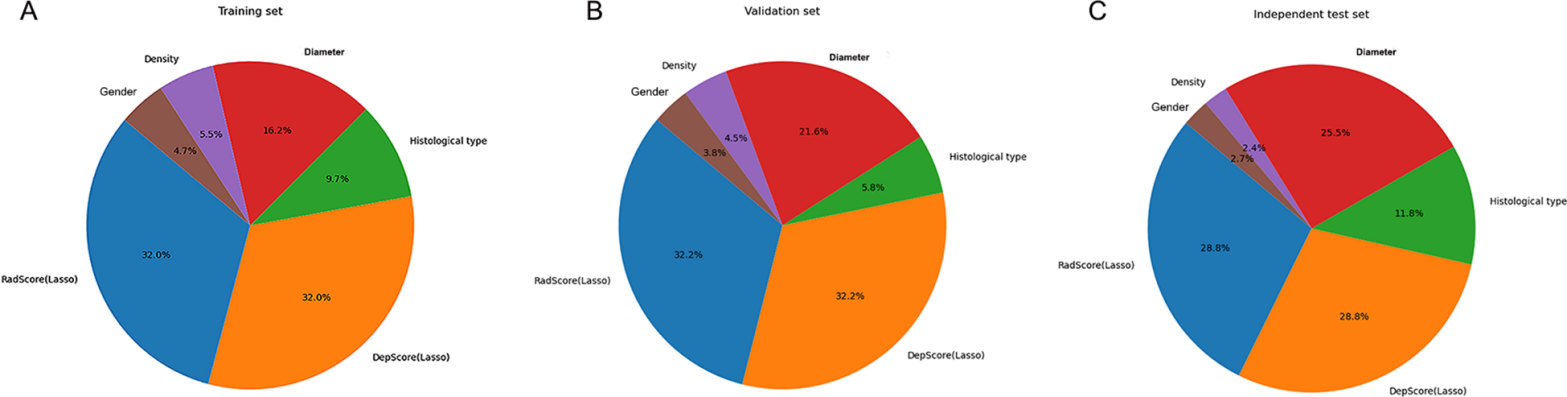
The relative contribution of features in the training **(A)**, validation **(B)**, and independent test **(C)** sets.

The combined model performance metrics revealed AUC values of 0.929 in the training set, 0.825 in the validation set, and 0.892 in the independent test set (**Table 3**, **Figures 4A–C**). In the independent test set, the combined model demonstrated significant improvements in overall predictive performance, with IDI values of 0.115, 0.288, and 0.095 over the clinical-radiological, radiomics, and deep learning models, respectively (**Table 4**). The DCA demonstrated that the combined model had a superior clinical net benefit than all the other models (**Figures 4D–F**). The calibration curves revealed that the combined model displayed strong agreement between predictions and pathology results (**Figures 4G–I**), with Brier scores of 0.101 (training set), 0187 (validation set) and 0.125 (independent test set), indicating excellent probabilistic calibration. Among all the features, deep-score and rad-score contributed the most to the prediction (**Figures 5A–C**).

**Table 4:** Comparison of predictive performance among all models.

Model	Data set	IDI (95% CI)	*P* (IDI)
Combined *vs* Clinical-radiological	Training	0.322 (0.244-0.399)	<0.001
	Validation	0.144 (0.039-0.249)	0.007
	Independent test	0.115 (-0.014-0.243)	0.080
Combined *vs* Radiomics	Training	0.303 (0.244-0.361)	<0.001
	Validation	0.156 (0.048-0.265)	0.005
	Independent test	0.288 (0.175-0.401)	<0.001
Combined *vs* Deep learning	Training	0.044 (0.010-0.077)	0.011
	Validation	0.025 (-0.034-0.084)	0.405
	Independent test	0.095 (0.038-0.152)	0.001

### 3.5 Model visualization and interpretation

**Figure 6:**
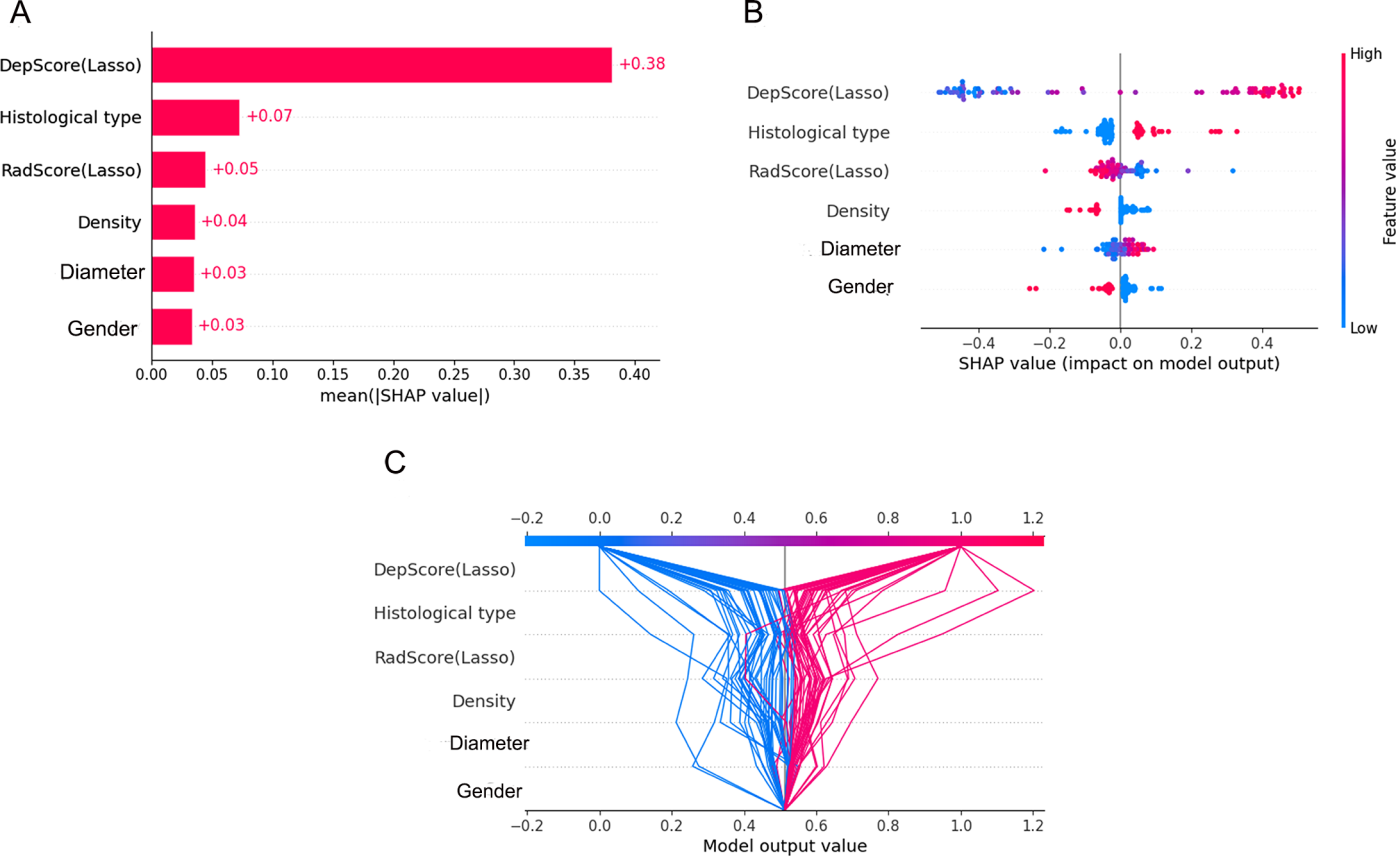
Global visualization plots. **(A–C)** represent the feature importance plot, swarm plot, and decision plot, respectively.

For global feature analysis: the importance plot (**Figure 6A**) showed the relative significance ranking of features, with Deep-score being the most influential; the bee-swarm plot (**Figure 6B**) displayed SHAP values for each sample across features (positive values increased the probability of high Ki-67 expression, while negative values reduced it); the decision plot (**Figure 6C**) illustrated cumulative feature impacts on model predictions, with gray lines denoting baseline values, red trajectories mapping positive samples (high-expression group), and blue trajectories tracking negative samples (low-expression group).

**Figure 7:**
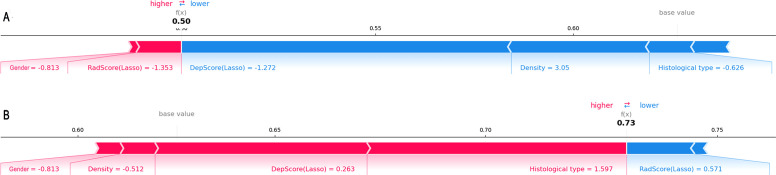
Force plots illustrating individual predictions. Shown are two representative samples from the low-expression group **(A)** and the high-expression group **(B)**.

For individual visualization, force plots (**Figures 7A, B**) illustrated the model’s decision-making process for both high- and low-expression cases. Score calculation started from the baseline value, with each feature represented as a directional force via SHAP values. Feature contributions were quantified by colored bars: bar length corresponded proportionally to the feature’s influence magnitude on the final prediction value f(x). Specifically, red bars indicated increased probability of high expression, while blue bars denoted decreased probability. These directional forces were then cumulatively aggregated to yield the overall effect.

## 4 Discussion

In this study, we developed a combined model integrating clinical-radiological, radiomics, and deep learning features to non-invasively characterize intra-heterogeneity and predict Ki-67 expression level in NSCLC. Our findings demonstrate that the combined model achieved superior predictive performance compared to single models, maintaining robust generalizability with an AUC of 0.892 in the independent test set. SHAP analysis elucidated the combined model’s decision-making process, reinforcing its clinical credibility and generalizability. This study is anticipated to enable clinicians to preoperatively evaluate tumor invasiveness and optimize personalized therapeutic strategies.

NSCLC with different Ki-67 expression levels exhibits distinct biological behaviors and gene expression patterns. These visually imperceptible “differences” can be captured via medical imaging and quantified through high-throughput feature extraction, thereby clarifying the association between these features and the underlying pathophysiological processes (31, 32). Previous studies have explored the predictive value of CT-derived radiomics features for Ki-67 expression levels, with test set AUCs ranging from 0.77–0.84 (15, 27, 33). However, conventional radiomics features predominantly rely on mathematical formulations and are susceptible to technical variabilities, such as image noise, manual segmentation variability, and CT scan parameters (34). In contrast, deep learning algorithms excel at autonomously learning high-level abstract representations from images, effectively overcoming the limitations of handcrafted features. This advantage has driven their widespread use in recent medical research (20, 35). Building on these foundations, our study leverages the ResNet18 convolutional neural network to extract deep learning features from CT images for Ki-67 prediction. More importantly, we integrate diverse feature types through Machine Learning to achieve comprehensive multi-modal information fusion. The combined model demonstrated significantly enhanced predictive performance, underscoring the complementary value of multi-modal features. Liu et al. (36) fused radiomics and deep learning features from DCE-MRI images, and similarly found that the fusion improved the accuracy of preoperative lymph node metastasis prediction in breast cancer. Kim et al. (37) combined deep learning and radiomics features extracted from CT images to predict the epidermal growth factor receptor mutation status in NSCLC patients; results showed this combination of radiomics and deep learning was feasible.

In clinical practice, lung cancer treatment decisions should rely on robust evidence rather than algorithm-generated probabilities. This underscores the critical need to address interpretability challenges inherent in machine learning’s “black-box modeling.” Our study used SHAP technology to enhance the combined model’s interpretability, providing transparency into feature contributions and the model’s decision-making process. Results showed significantly higher deep-score and rad-score in the high-Ki-67 expression group than the low-expression group, consistent with previous research (27). Deep-score and rad-score are typically positively correlated with tumor heterogeneity (38). NSCLC with high Ki-67 expression exhibits stronger proliferative, infiltrative, and invasive abilities, resulting in more complex intra-tumoral texture and greater heterogeneity (9, 39). The high-Ki-67 expression group primarily included males, squamous cell carcinoma cases, and solid tumors with larger diameters, consistent with previous research (28, 40). Warth et al. (41) evaluated Ki-67 expression in three large independent NSCLC cohorts (total n=1,065) and found that squamous cell carcinoma had a mean expression level (52.8%) twice that of adenocarcinoma (25.8%). A meta-analysis further confirmed higher Ki-67 expression in squamous cell carcinoma than in adenocarcinoma (7). Additionally, more solid tumor components and larger diameters indicate more aggressive tumor biology, hence higher Ki-67 expression compared to non-solid tumors and smaller lesions (27, 42). Previous studies reported that CT radiological features (e.g., lobulation, vacuole or cystic component) showed significant differences across Ki-67 expression levels, but findings were inconsistent (15, 43). In the present study, no such differences were observed between the high- and low-expression groups. This discrepancy may stem from two key limitations: Firstly, physicians can only obtain limited valuable information from CT radiological features via visual inspection. Secondly, evaluation of these features often relies on physicians’ subjective judgment and clinical experience, leading to poor consistency and reproducibility that introduces bias into research findings.

There were some limitations to this study. First, this study has a retrospective design with an inherent risk of selection bias, and the sample size, though validated in an external set, remains limited. Therefore, the findings should be interpreted as proof-of-concept, and prospective validation in a larger, multi-center cohort is warranted before clinical application. Second, our analysis focused exclusively on intra-tumoral features, and the predictive value of peritumoral regions warrants future investigation. Third, data from enhanced CT were not included in this study. Because previous studies had suggested that high-density contrast in enhanced images may mask the original textural features of the lesion tissue (44). And enhanced CT scanning also carried the risk of iodine contrast use and renal burden. However, it was also noted that radiomics features based on biphasic enhanced CT images could predict Ki-67 expression level (27). Therefore, the inclusion of data from enhanced scans needs to be further explored. Finally, future work will aim to incorporate multi-modal data (e.g., pathomics, transcriptomics) to improve the biological interpretability of the models.

## 5 Conclusion

This study developed and validated an interpretable model combining clinical-radiological, radiomic, and deep learning features for the non-invasive prediction of Ki-67 expression levels. This model is expected to support clinicians in the early assessment of tumor proliferation activity in patients with NSCLC, providing complementary information to inform personalized treatment strategies.

## Data Availability

The raw data supporting the conclusions of this article will be made available by the authors, without undue reservation.
