# Supplementary material for: Predicting Ki-67 expression levels in non-small cell lung cancer using an explainable CT-based deep learning radiomics model
Source: Front Oncol. 2025 Dec 10;15:1655714. doi: 10.3389/fonc.2025.1655714 (PMC12727595; doi:10.3389/fonc.2025.1655714)
Supplement: Supplementary file 1 [file Table1.docx]

Supplementary Table S1 Comparative performance of different classifiers in the combined model

| Classifier | Training cohort AUC (95%CI) | Validation cohort AUC (95%CI) |
| --- | --- | --- |
| Decision Tree | 0.967 (0.947-0.987) | 0.819 (0.727-0.912) |
| Random Forest | 0.937 (0.903-0.970) | 0.816 (0.719-0.913) |
| XGBoost | 1.000 (1.000-1.000) | 0.830 (0.738-0.921) |
| Logistic Regression | 0.835 (0.778-0.893) | 0.778 (0.671-0.884) |
| **Support Vector Machine** | **0.929 (0.892-0.966)** | **0.825 (0.733-0.917)** |
