# Supplementary material for: Predicting Ki-67 expression levels in non-small cell lung cancer using an explainable CT-based deep learning radiomics model
Source: Front Oncol. 2025 Dec 10;15:1655714. doi: 10.3389/fonc.2025.1655714 (PMC12727595; doi:10.3389/fonc.2025.1655714)
Supplement: Supplementary file 2 [file Table2.docx]

| Model | Classifier | Regularization parameter (C) | Kernel coefficient (Gamma) | Kernel function |
| --- | --- | --- | --- | --- |
| Clinical-radiological | Support vector machine | 1.0 | 0.01 | sigmoid |
| Radiomics | Support vector machine | 8.0 | 0.1 | rbf |
| Deep learning | Support vector machine | 20.0 | 0.1 | rbf |
| Combined | Support vector machine | 8.0 | 0.01 | sigmoid |

Supplementary Table S2 Parameters of the models
