# Supplementary material for: Predicting Ki-67 expression levels in non-small cell lung cancer using an explainable CT-based deep learning radiomics model
Source: Front Oncol. 2025 Dec 10;15:1655714. doi: 10.3389/fonc.2025.1655714 (PMC12727595; doi:10.3389/fonc.2025.1655714)
Supplement: Supplementary file 3 [file Table3.docx]

Supplementary Table S3 Radiomic features selected by LASSO and their coefficients

| Type | Filter | Features | LASSO coefficient |
| --- | --- | --- | --- |
| Glrlm | Wavelet_LLH | RunVariance | 0.126 |
| Gldm | Wavelet_LLL | LargeDependenceHighGrayLevelEmphasis | 0.054 |
| Gldm | Boxmean | LargeDependenceHighGrayLevelEmphasis | 0.049 |
| Glrlm | Wavelet_LLL | LongRunHighGrayLevelEmphasis | 0.026 |

Rad-score=0.126184627×wavelet_glrlm_wavelet-LLH-RunVariance+0.0541923419×wavelet_gldm_wavelet-LLL-LargeDependenceHighGrayLevelEmphasis+0.0489606448×boxmean_gldm_LargeDependenceHighGrayLevelEmphasis+0.0256320611×wavelet_glrlm_wavelet-LLL-LongRunHighGrayLevelEmphasis+0.4198895
