# Supplementary material for: Predicting Ki-67 expression levels in non-small cell lung cancer using an explainable CT-based deep learning radiomics model
Source: Front Oncol. 2025 Dec 10;15:1655714. doi: 10.3389/fonc.2025.1655714 (PMC12727595; doi:10.3389/fonc.2025.1655714)
Supplement: Supplementary file 4 [file Table4.docx]

Supplementary Table S4 Deep leanring features selected by LASSO and their coefficients

| Features | LASSO coefficient |
| --- | --- |
| Deep_feature121 | 0.301 |
| Deep_feature143 | 0.011 |
| Deep_feature2 | -0.020 |
| Deep_feature49 | -0.039 |

Deep-score=0.3014318×Deep_feature121+0.0110030482×Deep_feature143+-0.0203756448 ×Deep_feature2+-0.0392154939×Deep_feature49 + 0.4198895
