# Supplementary material for: Predicting Ki-67 expression levels in non-small cell lung cancer using an explainable CT-based deep learning radiomics model
Source: Front Oncol. 2025 Dec 10;15:1655714. doi: 10.3389/fonc.2025.1655714 (PMC12727595; doi:10.3389/fonc.2025.1655714)
Supplement: Supplementary file 5 [file DataSheet1.docx]

**2.6.2 Deep learning Feature extraction and selection**

The original CT images underwent multi-stage preprocessing resampling to a median voxel spacing of 0.7×0.7×1 mm³ (trilinear interpolation for images and nearest-neighbor interpolation for corresponding VOIs), extraction of random 64×64×48 patches from annotated VOIs, and data augmentation via geometric transformations (flipping, rotation, scaling, translation) to enhance diversity and prevent overfitting. Finally, voxel values were normalized via windowing (width: 1500 HU, level: -600 HU).
