# Supplementary material for: Predicting Ki-67 expression levels in non-small cell lung cancer using an explainable CT-based deep learning radiomics model
Source: Front Oncol. 2025 Dec 10;15:1655714. doi: 10.3389/fonc.2025.1655714 (PMC12727595; doi:10.3389/fonc.2025.1655714)
Supplement: Supplementary file 6 [file DataSheet2.docx]

1. **Results**

3.3 **Prediction performance of the deep learning model**

To enhance the interpretability of the feature extraction process, this study incorporated a CAM module into the deep learning network (1). This technique generates intuitive heatmaps by leveraging the feature maps from the final convolutional layer and the corresponding weights. As illustrated in Figure 3, these heatmaps highlight the areas within the input CT images that the deep learning network prioritized during feature encoding. The red-highlighted areas correspond to the critical regions upon which the network focused, specifically the central tumor region. This visualization helps verify whether the network bases its feature representations on clinically relevant lesion features rather than irrelevant anatomical structures, thereby increasing the transparency and trustworthiness of the overall pipeline in a clinical diagnostic context.

**REFERENCES**

1. Zhang Y, Hong D, McClement D, Oladosu O, Pridham G, Slaney G. Grad-CAM helps interpret the deep learning models trained to classify multiple sclerosis types using clinical brain magnetic resonance imaging. J Neurosci Methods. (2021) Apr 1;353:109098. doi: 10.1016/j.jneumeth.2021.109098.
